# Draft Genome Sequence of the Anti-Algal Marine Actinomycete *Streptomyces* sp. JS01

**DOI:** 10.1128/genomeA.01261-14

**Published:** 2014-12-04

**Authors:** Huajun Zhang, Su Zhang, Yun Peng, Yi Li, Zhangran Chen, Wei Zheng, Hong Xu, Zhiming Yu, Tianling Zheng

**Affiliations:** a State Key Laboratory of Marine Environmental Science and Key Laboratory of the Ministry of Education for Coastal and Wetland Ecosystems, School of Life Sciences, Xiamen University, Xiamen, China; b Key Laboratory of Marine Ecology and Environmental Science, Institute of Oceanology, Chinese Academy of Sciences, Qingdao, China

## Abstract

*Streptomyces* sp. JS01 is the producer of an anti-algal compound that shows inhibitory activity against a harmful algal species *Phaeocystis globosa* and can also produce a red pigment. Its genome sequence will allow for the characterization of the anti-algal compound and the molecular mechanisms underlying its beneficial properties.

## GENOME ANNOUNCEMENT

Harmful algal blooms (HABs), one of the results of eutrophication, break the balance of the marine ecosystem and cause severe damage to the marine environment, threatening other organisms and even human health (1, 2). With the aim of terminating HABs, several approaches including physical and chemical methods have been carried out (3–6). Studies on the relationship between algae and bacteria have resulted in the isolation of actinomycetes capable of inhibiting or killing HAB species. *Streptomyces* sp. JS01 showed a high inhibitory effect on a harmful algal species, *Phaeocystis globosa*, that we isolated from mangrove sediment. *Streptomyces* sp. JS01 was deposited in the Marin Culture Collection of China [MCCC] under the collection no. MCCC 1F01225. Here, we report the genome sequence of *Streptomyces* sp. JS01 and try to identify the anti-algal compound and its biosynthetic gene clusters. The genome information may provide a basis for further research directly related to the compound product synthesis field.

The genome was sequenced using an Illumina HiSeq instrument at the Beijing Novogene company (Beijing, China). A library containing 500-bp inserts was constructed. After the sequencing runs, it produced about 1.46-Gbp raw data, representing 145-fold coverage of the genome. The sequences were assembled into 38 contigs using SOAPdenovo software, the largest being 2,010,013 nucleotides and the shortest, 511 nucleotides. The initial draft genome size was 7.8 Mb, with a G+C content of 71.6%. Genome assembly was annotated using NCBI Prokaryotic Genome Annotation Pipeline (released 2013) and RAST version 2.0 (7), resulting in the identification of 6,806 genes, 7 rRNA operons, and 71 tRNA loci. RAST-based annotation revealed that JS01 has a high ability to cope with copper stress because the coding genes for copper homeostasis (*n* = 12) and copper transport systems (*n* = 13) highlighted it. The genetic presence of cobalt, zinc, and cadmium (*n* = 13) can also help JS01 to resist heavy metals. The arsenic resistance genes *ars*R, *ars*A, *ars*B, and *ars*C and the ACR3 protein were also observed in the draft genome. JS01 also has the potential to resist some antibiotics such as streptothricin (*n* = 1) and fluoroquinolones (*n* = 5). However, the compound responsible for its anti-algal activity was not discovered. The analysis of the draft genome sequence of JS01 coupled with already existing genomes of anti-algal strains of the genus might provide more information to aid in the search for the anti-algal related genetics.

### Nucleotide sequence accession numbers.

This whole-genome shotgun project has been deposited at DDBJ/EMBL/GenBank under the accession no. JPWW00000000. The version described in this paper is the first version, JPWW01000000.
